# Local and Regional Therapies for Hepatocellular Carcinoma and Future Combinations

**DOI:** 10.3390/cancers14102469

**Published:** 2022-05-17

**Authors:** Adam Hatzidakis, Lukas Müller, Miltiadis Krokidis, Roman Kloeckner

**Affiliations:** 1 Department of Radiology, AHEPA University Hospital of Thessaloniki, Faculty of Health Sciences, School of Medicine, Aristotle University of Thessaloniki, 54124 Thessaloniki, Greece; 2 Department of Diagnostic and Interventional Radiology, University Medical Center of the Johannes Gutenberg University Mainz, 55131 Mainz, Germany; lukas.mueller@unimedizin-mainz.de (L.M.); roman.kloeckner@unimedizin-mainz.de (R.K.); 3 1st Department of Radiology, Areteion Hospital, School of Medicine, National and Kapodistrian University of Athens, 11528 Athens, Greece; mkrokidis@hotmail.com

**Keywords:** hepatocellular carcinoma, percutaneous treatment, locoregional treatment, chemoembolization, tumor ablation, radioembolization, immunotherapy

## Abstract

**Simple Summary:**

Percutaneous interventional radiological techniques offer many alternatives for treatment of Hepatocellular Carcinoma (HCC) using local anesthesia and sedation. These methods aim to destroy the malignant tumors locally without affecting the non-malignant liver. In this way, complications are kept low and patient recovery is quick. Indications depend on tumor size, type and stage, as well as patient’s condition, liver function and co-morbidities. In recent years, a lot of research has been made in combining such approaches with immune therapy, but there is still much work to be done. This manuscript tries to analyze where we stand today and explain, using a comprehensive algorithm, the treatment options for each different clinical condition.

**Abstract:**

Background: Hepatocellular carcinoma (HCC) can be treated by local and regional methods of percutaneous interventional radiological techniques. Indications depend on tumor size, type and stage, as well as patient’s condition, liver function and co-morbidities. According to international classification systems such as Barcelona Clinic Liver Cancer (BCLC) classification, very early, early or intermediate staged tumors can be treated either with ablative methods or with transarterial chemoembolization (TACE), depending on tumor characteristics. The combination of both allows for individualized forms of treatment with the ultimate goal of improving response and survival. In recent years, a lot of research has been carried out in combining locoregional approaches with immune therapy. Although recent developments in systemic treatment, especially immunotherapy, seem quite promising and have expanded possible combined treatment options, there is still not enough evidence in their favor. The aim of this review is to provide a comprehensive up-to-date overview of all these techniques, explaining indications, contraindications, technical problems, outcomes, results and complications. Moreover, combinations of percutaneous treatment with each other or with immunotherapy and future options will be discussed. Use of all those methods as down-staging or bridging solutions until surgery or transplantation are taken into consideration will also be reviewed. Conclusion: Local and regional therapies remain a mainstay of curative and palliative treatment of patients with HCC. Currently, evidence on potential combination of the local and regional treatment options with each other as well as with other treatment modalities is growing and has the potential to further individualize HCC therapy. To identify the most suitable treatment option out of these new various options, a repeated interdisciplinary discussion of each case by the tumor board is of utmost importance.

## 1. Transarterial Chemoembolization (TACE)

### 1.1. Background

According to the current EASL guidelines, TACE is the recommended first-line treatment for patients within the intermediate stage, which is defined as multinodular tumor burden, preserved liver function and good performance status [1]. The recent 2022 update of the BCLC criteria limits the recommendation for TACE to patients with well-defined tumor nodules, preserved portal flow and selective vascular access (namely the second subgroup of BCLC stage B), that are not eligible for liver transplantation with extended Milan criteria (first subgroup of BCLC stage B) [2]. Following these recommendations, patients with diffuse, infiltrative and extensive bilobar liver involvement do not benefit from TACE (third subgroup of BCLC stage B patients), and should be better candidates for systemic treatment in the first line. However, no clear cut-off criteria when to prefer systemic treatment over TACE can be provided up to date [2]. The suggestions for TACE as recommended therapy in intermediate stage mainly rely on two randomized trials, which showed a survival benefit of TACE compared to best supportive care [3,4]. However, patient selection in both trials followed very strict inclusion criteria. In clinical reality, the intermediate stage comprises a heterogeneous subgroup of patients with distinct differences in tumor burden and remaining liver function [5]. Furthermore, the concept of stage-migration is commonly applied, meaning that patients in earlier or more advanced stages are referred to TACE according to individual treatment concepts.

### 1.2. Biological Rational for TACE

Chemoembolization relies on important tumor characteristics of HCC: the tumor tissue has, in most cases, a strong arterial tumor supply, which is based on an intense neo-angiogenesis during tumor development and progression. Embolization takes advantage of HCC’s strong arterial supply, aiming for complete anoxia inside the malignancy, therefore inducing an ischemic reaction and leading to tumor necrosis. Theoretically, the surrounding liver parenchyma is spared out from these necrotic effects as it receives its blood supply mainly from the portal venous system. However, the most peripheral part of the tumor nodules does receive blood supply from the branches of the portal vein. Thus, there is always a rest supply of the peripheral tumor that could not be stopped with TACE. Therefore, TACE is not considered as curative treatment option in patients with HCC. With the additional local application of chemotherapeutic agents (most commonly the anthracyclines doxorubicin and epirubicin or the platinum-derivates cisplatin or miriplatin), TACE has theoretically the benefit of a synergistic effect with a high local chemotherapy concentration leading to a higher rate of tumor cell necrosis.

### 1.3. Technical Considerations

Up to date, two main TACE techniques are the standard of care: The conventional TACE (cTACE) was already used before 1990 as injection of an emulsion consisting of chemotherapy and lipiodol followed by an embolizing agent through diagnostic catheters and was further developed after 2000 as a superselective embolization technique with the use of flexible microcatheters [3]. In 2006, drug-eluting beads TACE (DEB-TACE) was introduced, which is based on a slower release of the chemotherapeutic agents to improve its therapeutic effect while reducing side effects [6]. Since then, several trials compared the outcome of both TACE types [7,8,9,10]. However, no significant difference has been observed regarding tumor response or survival. Both types only differed regarding post-procedural pain and chemotherapy-associated systemic side effects, which were less often observed in patients undergoing DEB-TACE [7,10]. Regardless of the applied chemotherapeutic agent, TACE should be performed in a superselective manner, as this leads to an increased rate of tumor necrosis and minimizes the damage in the surrounding liver tissue [11,12].

### 1.4. Patient Selection

In more than 80% percent of the cases, HCC develops in a cirrhotic liver [1]. Thus, the patients suffer from a combination of two chronic illnesses: progressing liver damage resulting in an impaired synthesis function and the HCC tumor burden itself. Therefore, the rationale for initiation and continuation should be based on both the tumor and the remaining liver function.

### 1.5. Patient Selection: Tumor Burden

Regarding the tumor burden, the image-derived features tumor size and tumor number correlate with the prognosis [1,4,13,14,15,16]. Furthermore, a large tumor size is related to a higher risk for complications [12,16,17,18,19]. Although the current guidelines do not recommend a clear cut-off regarding the tumor size, in clinical reality, a commonly used cut-off is a single nodule size of 5 cm as a response to TACE is significantly lower if this threshold is exceeded [12]. Furthermore, a total tumor burden of more than half of the total liver volume is a contraindication for TACE as radiologic response is unlikely and the risk of post-interventional hepatic decompensation is substantially increased [1]. Several systems for the stratification of patients according to the sum of tumor size and the number of nodules have been proposed [20,21,22]. However, their prognostic value in external validation was only moderate and a direct head-to-head comparison of the various scores and cut-offs is missing. Apart from tumor size, number and growth type, pre-procedural imaging offers an insight into various prognosis-related tumor features [23]. One of the most important features is the degree and anatomy of tumor vascularity, which highly influences the treatment success. Furthermore, the growth pattern of the tumor correlates with the biological aggressiveness and infiltrative and diffuse tumor growth is linked to an impaired OS [24,25]. Concretely, the current guidelines recommend a critical discussion on the indication for TACE in patients with severe hepatic decompensation (Child-Pugh B with signs of decompensation and Child-Pugh C) as those are at high risk for post-interventional liver failure. Furthermore, patients with bilirubin levels above 2 mg/dl have a high potential for post-interventional hepatic decompensation [1,2].

### 1.6. Patient Selection: Remaining Liver Function

Regarding the liver function, particularly the albumin-bilirubin (ALBI) score has gained importance as an easy-to-use estimate of the patients’ remaining liver function in the recent decade [26,27]. In comparison to the Child-Pugh score, the ALBI score does not contain subjectively estimated parameters such as the degree of ascites or the degree of encephalopathy and is only composed of objective laboratory parameters. Due to the high predictive performance for patients undergoing TACE, the ALBI score, as well as other liver function-related parameters such as INR, platelet count and cholinesterase play an important role in treatment decision making [28,29,30]. Despite laboratory parameters, other factors related to an impaired liver function such as, e.g., ascites or a reduction in the overall patient status should be considered in decision-making. The presence of other surrogates for an elevated portal pressure, however, seem to play a minor role in the initial patient selection [31].

### 1.7. Unmet Problems

Although extensively evaluated, several problems prior to and during TACE treatment remain unclear. One common problem is the planning of re-treatment in patients with remaining vital tumor after the initial TACE. Up to date, it remains unclear whether an on-demand treatment repetition or a fixed schedule is superior. Scheduled treatment in regular intervals may lead to improved patient compliance and monitoring of the case, but an aggressive schedule may also increase the risk of liver failure [32]. Thus, liver function and performance status have to be monitored closely in order to avoid “overtreatment” [33]. From our point of view, regardless of the re-treatment type, continuous re-evaluation of each patient by an interdisciplinary tumor board is of the utmost importance [23]. Each indication should be jointly approved by this board.

Another common problem during follow-up is the optimal time-point to switch to other treatment modalities in case of tumor progress and/or ongoing decrease in the remaining liver function. In clinical routine, the optimal time-point for a therapy switch may be hard to determine, particularly as defined cut-off criteria are missing [23].

Additional TACE sessions over a certain point may not lead to a survival benefit for the patient, while delaying the switch to systemic therapy or even completely impeding this switch due to a deterioration of the remaining liver function caused by repetitive TACE could lead to an impaired survival outcome [34,35,36,37,38]. One prospective trial currently investigating the role of systemic treatment following TACE is the OPTIMIS trial (NCT01933945). In cases of tumor progression, decrease in remaining liver function, early recurrence, incomplete necrosis and occurrence of extrahepatic spread and/or vascular invasion during TACE, but also in the case of unbearable side effects (e.g., severe forms of post-embolization syndrome or hepatorenal syndrome), TACE treatment should be immediately stopped.

### 1.8. Long-Term Outcome and Risk Prediction

For patients with intermediate-stage HCC, a median OS of 2.5 years can be expected [1]. A median OS of up 4 years can be achieved in case of strict patient selection [13]. However, especially because of the above-mentioned heterogeneity of patients undergoing TACE in conjunction with the concept of stage migration, the individual prognosis prediction remains difficult. Although several risk scores for patient selection prior to or during TACE have shown promising results initially, all showed only moderate performance in external validation [19,21,23,28,39,40,41,42,43,44,45,46,47,48,49,50,51,52,53,54]. The underperformance of these scoring systems might not only be explained through the heterogeneity of the patient cohort, but also by the complex interplay of co-existing liver cirrhosis and HCC as two synchronous diseases. All these issues are reasons why none of the scoring systems play a significant role in clinical reality—although the need for precise risk estimation is tremendous.

Thus, current attempts try to include novel risk factors to improve the predictive performance of risk scoring systems. In particular, the knowledge on the interplay of tumor development and progression as well as immune response and inflammatory reaction has been growing continuously [55,56,57,58,59]. Especially the role of neutrophils for HCC development and progression on the tumor microenvironment as well as in systemic reaction is under investigation. Moreover, the relations of neutrophils to lymphocytes and platelets to lymphocytes, namely NLR and PLR, have been identified as important prognostic marker in HCC and particularly in patients undergoing TACE [58,59,60,61,62,63,64]. Based on the first experiences regarding the potential of immune response and inflammation, several other indices based on various laboratory markers have been investigated in patients with HCC undergoing TACE (Table 1).

Furthermore, current achievements in the field of AI-based risk prediction automatically allow the inclusion of a great number of risk factors simultaneously. First studies have shown the feasibility of this approach for patients with HCC undergoing TACE, which outperformed conventional risk scoring distinctly [69].

### 1.9. Combination of TACE and Thermal Tumor Ablation

One treatment combination, which has been extensively investigated in recent years, is the combination of TACE and thermal tumor ablation. Such a combination might lead to a reduction of the TACE-induced neo-angiogenesis and therefore reduces the risk of tumor recurrence and metastatic growth [70]. Furthermore, the combination of radiofrequency ablation and TACE increases the coagulated zone, which led to a significantly reduced rate of local tumor progression [71,72]. For patients within the early tumor stage, several meta-analyses showed a survival benefit and a better regression-free survival when combining TACE and ablation in patients unsuitable for resection [73,74,75]. Specifically, patients with a large tumor size could benefit [76,77,78]. For patients with intermediate-stage HCC, ablation might be a suitable addition in selected patients with a favorable tumor location [70]. Liu et al. suggest that for HCCs > 5 cm combination of both methods could lead to better outcome results [75]. They propose that for such large tumors, first-line TACE followed by ablation 1 month later is better than TACE alone. However, clear evidence on the combination of TACE and ablation is, particularly for Western patients, missing [1].

### 1.10. Combination of TACE and TARE

Studies investigating the sequential combination of transarterial radioembolization (TARE) with TACE are rare. Comparative analysis and RCTs on the sequential or parallel use of both techniques are missing. Preliminary results, however, indicate an acceptable safety profile and good treatment effect for specific patient conditions [79]. Through a different biologic rationale in comparison to TACE, TARE might be particularly an alternative to systemic treatment in patients with extensive liver progress during TACE.

### 1.11. Combination of TACE and SBRT

Another option for patients with unresectable HCC is the combination of TACE and stereotactic body radiotherapy (SBRT). SBRT is particularly effective in tumor areas with high oxygenation namely the tumor periphery, where TACE itself is less effective [80]. On the other hand, cytotoxic agents used for TACE could lead to a higher radiosensitivity [81]. A recent meta-analysis yielded a prolonged survival and an improved response for the combination of SBRT and TACE in comparison to SBRT alone for unresectable HCC. However, no survival benefit was observed for patients with portal vein tumor thrombosis (PVTT). Contrary, results of a phase 2 trial (NCT01901692), which compared combined SBRT and TACE against sorafenib for patients with macroscopic vascular invasion, showed a significant survival benefit for patients treated with SBRT and TACE [82]. Currently, several phase 3 RCTs on the combination of TACE and external radiotherapy for patients with unresectable HCC are recruiting (NCT03116984, NCT02794337, NCT03939845). Up to date, no recommendation can be made towards a generalized use for the combination of TACE with internal (TARE) or external (SBRT) radiotherapy for patients with unresectable HCC.

### 1.12. Combination of TACE with Systemic Treatment Agents and Future Directions

Multiple trials have investigated the combination of TACE and sorafenib and other tyrosine-kinase inhibitors. Recently, the TACTICS trial was the first to show an improved progression-free survival (PFS), while other trials have consistently failed to show a significant survival benefit [83,84,85,86,87,88,89]. The results of the TACTICS trial, which included intermediate-stage HCC patients, however, have to be interpreted with the background of its specific study characteristics [84]. In comparison to previous studies, the patients included in the TACTICS trial had mostly a better remaining liver function and in most cases no pre-existing liver cirrhosis [90]. Furthermore, the response was assessed using the RECICL criteria and not the commonly used mRECIST or RECIST1.1 criteria. These RECICL do not define new intrahepatic lesions as progressive disease and therefore not as an endpoint for PFS, which is rather special. Furthermore, a recent post-hoc analysis yielded no differences in the overall survival [83]. Thus, through the unconventional methodology in combination with the lacking survival benefit, no treatment recommendations can be made based on the results of the TACTICS trial for intermediate-stage HCC.

Currently, there are a few phase 2/3 trials investigating the combination of TACE and immunotherapeutic agents [91] (Table 2).

Theoretically, locoregional treatment might be the perfect partner for immunotherapy. Tumor necrosis induced through TACE might lead to a release of tumor-associated antigens, which could activate the tumor microenvironment and stimulate the specific immune response [11,92]. This could enhance the effect of immunotherapy agents programming the immune system against cancer cells again [93]. Furthermore, TACE-induced hypoxia increases the production of vascular endothelial growth factor (VEGF), which catalyzes recurrent tumor growth due to an increase in re-vascularization [94,95,96]. VEGF inhibitors could play a counterpart and inhibit the re-vascularization [93]. However, up to date, optimal patient selection remains difficult as evidence for biomarkers in patients with HCC and immunotherapy is low and has only been evaluated in small, retrospective studies. Particularly evidence on biomarkers in combined locoregional treatment and immunotherapy is scarce [97,98]. Besides the RCTs currently focusing on the combination of TACE and immunotherapy, the ABC-HCC trial (NCT04803994) is comparing TACE versus AtezoBev head-to-head and therefore investigating this promising combination which has become standard of care for patients within the advanced stage after the positive IMBRAVE150 results [2,99]. The RENOTACE (NCT04777851) trial is a second RCT on immunotherapy (regorafenib + nivolumab) versus TACE in the intermediate stage. However, this trial has not started recruitment yet.

### 1.13. Current Recommendations

In summary, the recommendations for patients with intermediate-stage HCC rely on two phase 3 RCTs [3,4]. Based on these results and those of several meta-analyses, the current guidelines strongly recommend TACE in patients within the intermediate stage [1]. Two RCTs and one meta-analysis compared cTACE and DEB-TACE but did not find significant outcome differences. Thus, there is strong evidence that neither technique has to be favored [1]. All of the other recommendations in patients with HCC undergoing TACE, particularly on possible prognostic factors, are mainly based on retrospective studies.

## 2. Transarterial Radioembolization (TARE)

### 2.1. Background

Transarterial radioembolization (TARE), also known as selective internal radiation therapy (SIRT), is based on an application of radioactive particles directly into the liver artery. The BCLC 2022 treatment scheme does not include TARE as standard-of-care for patients within the intermediate or the advanced stage [2]. However, TARE has been specifically named as a relevant alternative to tumor ablation and resection in patients with BCLC stage 0 and A (TARE could be considered in patients with single nodules <8 cm), based on the recent results of the LEGACY study, which indicates a prolonged duration of response and a clinically meaningful response rate for these patients [100]. The study included patients with single nodules less than 8 cm, Child-Pugh A and ECOG-PS 0/1. It is important to emphasize that the median tumor size of the patients included in that study was 2.6 cm (range 0.9–8.1). The current EASL guideline as well as the updated ESMO guideline both entitle TARE as an alternative treatment option in patients within early, intermediate and advanced stage [1,101]. However, no clear criteria for patient selection have been identified so far. Thus, the role of TARE in the treatment of unresectable HCC remains unclear and is mainly part of individual treatment concepts besides the standard recommendations.

### 2.2. Biological Rational for TARE

Similar to TACE, due to the higher proportion of arterial supply of the tumor tissue in comparison to the surrounding liver parenchyma, a high local concentration in the tumor tissue is intended. In comparison to TACE, however, the embolizing component is only minimal and the main effect of TARE is based on the radiation effect [102]. This absence of a vessel occlusion is of special importance in patients with portal venous occlusion. Under these circumstances, TARE might spare the remaining liver function and lower the risk for a post-procedural liver failure [1].

Prior to the actual TARE procedure, all patients have to undergo a pre-TARE angiography with application of technetium 99-labeled (^99^Tc) macroaggregated human albumin (MAA), which is followed by a SPECT/CT scan afterwards. This screening method is used to identify the patients with a relevant pulmonary shunt fraction not feasible for TARE. Furthermore, non-targeted vessels of the gastrointestinal tract can also be embolized during angiography to prevent gastrointestinal radiation damage [103]. Apart from TARE planning, the ^9^Tc-MAA-SPECT/CT yields important prognostic information for the post-interventional outcome: A recent subsequent analysis of the patients included in the SARAH trial showed a significant survival outcome benefit and a positive association with disease control in patients who had higher dose levels in the ^9^Tc-MAA-SPECT/CT [104]. However, estimation of the actual dose delivered to the tumor tissue during the TARE procedure remains difficult and is, in most cases, estimated using a combination of both, the body surface area of the patient as well as the hepatic tumor burden [105]. These dose calculations rely on a uniform blood supply of the tumor. However, most HCC lesions have an unequal, inhomogeneous tumor supply. Thus, a low dose could lead to undertreated tumor areas, while a high dose could cause damage of the surrounding liver parenchyma [103].

### 2.3. Patient Selection: TARE in Intermediate Stage

For patients within the intermediate stage, no results of large-scale randomized trials evaluating TACE vs.TARE are available [1]. Retrospective comparisons report less toxicity, better local tumor control and a longer progression-free survival as well as a better quality of life [106,107,108]. However, a survival benefit has not been observed so far, neither in retrospective comparisons nor in prospective pilot studies [109,110]. In comparison to TACE, TARE has several significant drawbacks: TARE requires a preprocedural angiographic evaluation, is less cost-effective and has a high personnel expenditure [103]. Thus, TARE has not become a standard procedure for patients within the intermediate stage. However, specifically in patients with a large tumor, for whom TACE is not recommendable due to a high risk of postembolization syndrome, TARE is a highly valuable treatment option. Furthermore, as the procedure relies on a different biological effect, TARE is a treatment option for patients with hepatic tumor progress after TACE. However, the role of TARE has not been fully defined yet. Thus, TARE as an individual treatment procedure after TACE failure requires an extensive interdisciplinary discussion.

### 2.4. Patient Selection: TARE in Advanced Stages

For patients within the advanced stage and particularly for patients with portal vein infiltration but without distant metastases, TARE has been considered a potential treatment option. Although benefits in quality of life and a better toxicity profile for TARE were reported, no survival benefit was observed in two phase 3 trials comparing TARE and sorafenib (SARAH trial and SIRveNIB trial) [111,112]. Up to date and similar to the intermediate stage, TARE remains an individual treatment option requiring an intense interdisciplinary evaluation. Furthermore, with immunotherapy as a novel treatment option for patients within the advanced stage, the role of TARE requires a re-definition supported by results of well-conducted RCTs.

### 2.5. Patient Selection: TARE for Bridging

For patients within the early stages, TARE is an option for bridging-to-transplant in selected patients. Initial clinical results indicate a better local tumor control leading to a higher transplantation rate in comparison to TACE [108]. However, the overall evidence is low and RCTs comparing TARE, TACE and ablation head-to-head are missing.

Besides bridging-to-transplant, the potential of TARE for downsizing has been reported in several studies. Downsizing through TARE in patients that initially did not meet the Milan criteria may lead to a tumor shrinkage enabling liver transplantation [113]. Furthermore, selective radioembolization could also facilitate a subsequent liver resection in patients with initially unresectable HCC as it causes hypertrophy of the future liver remnant [113].

### 2.6. Patient Selection: Radiation Segmentectomy

Furthermore, TARE has the potential to function as a curative treatment when performed in a specific manner: radiation segmentectomy, which is defined as highly selective TARE in one or two liver segments with a very high radiation dose. Radiation segmentectomy might serve as an additional treatment option in patients with a challenging tumor location, who are not amenable to thermal ablation or curative resection [114,115,116]. Furthermore, patients with comorbidities and limited remaining liver function could also benefit [116]. Initial clinical results in patients with a single HCC of 5 cm or smaller have been promising with a reported median OS between 4.4 and 6.5 years [115,116,117].

In summary, the few available RCTs evaluating TARE in intermediate stage and advanced stage did not find any survival benefit compared to standard treatment. Evidence on prognostic factors is low as it is mostly based on results from retrospective reports and meta-analyses as well as RCTs are missing. However, the current EASL guideline as well as the updated ESMO guideline do both entitle TARE as an alternative treatment option in patients within the early, intermediate and advanced stages. Thus, no clear recommendations and only weak suggestions on criteria for selecting patients likely to benefit from TARE can be made.

### 2.7. Combination of TARE with Other Locoregional Treatment Modalities

Evidence for the combination of TARE with TACE, SBRT and ablation is low. As mentioned above, the combination of TARE and TACE is scarcely investigated. The combination of TARE and SBRT in patients with portal vein infiltration seems to be safe and led to an improved prognosis [118,119]. However, these results are currently of experimental character and have been reported only for a very limited number of patients. Future validation is mandatory prior to clear recommendation.

### 2.8. Combination of TARE with Systemic Therapy and Future Directions

Due to its feasibility and beneficial toxicity profile in patients with high local tumor burden, TARE is potentially a combination partner to systemic treatment options. However, evidence for combined therapy is low. In the past, retrospective reports indicated a survival benefit for patients with an advanced-stage HCC treated with a combination of sorafenib and TARE in comparison to sorafenib alone [120]. However, the results of the phase 2 SORAMIC trial comparing sorafenib in combination with TARE versus sorafenib alone did not yield a survival benefit for the cohort undergoing combination therapy [121]. Nevertheless, post hoc subgroup analysis yielded a survival benefit for specific subgroups (patients without liver cirrhosis, nonalcoholic cirrhosis and patients younger than 65 years). Although promising, those subgroups were too small; therefore, additional trials are needed for clear recommendations [11]. Currently, the large phase 3 STOP-HCC trial with a similar design and the same treatment arms is recruiting (NCT01556490) [122].

Furthermore, several phase I and II trials on the combination of various immunotherapy agents and TARE are currently running [93]. Similar to other locoregional treatments, the release of tumor-related antigens during treatment could lead to a stimulation of the immune response [11]. This, again, could enhance the effect of immunotherapy agents programming the immune system against cancer cells [93]. As mentioned above, the locoregional treatment-induced hypoxia increases the production of vascular endothelial growth factor (VEGF), which catalyzes recurrent tumor growth due to an increase in re-vascularization [94,95,96]. Thus, VEGF inhibitors could antagonize this effect by impeding tumor re-vascularization. Apart from that, VEGF protects endothelial cells from radiation damage leading to a decrease in radio-sensitivity [93]. Thus, the blockade of VEGF potentially improves the response to radiation [93,123]. Therefore, VEGF inhibitors as a third component may further increase the duration of treatment response, ultimately leading to a survival benefit [93]. Hence, results of the currently recruiting trials are urgently awaited.

## 3. Ablation

### 3.1. Background

Chemical ablation for HCC has been historically performed by ethanol; however, this method has now been surpassed by energy-based ablation due to the significant recurrence rate [124,125]. Ethanol ablation as a loco-regional treatment method for small HCCs is nowadays replaced by thermal ablation. Ablation is an established treatment for HCC and, as described in the recently published BCLC guidelines [2], is the indicated treatment for the very early-stage lesions (BCLC 0: HCCs ≤ 2 cm, without vascular invasion or extrahepatic spread in patients with preserved liver function and no cancer-related symptoms). So, unless there is an option for liver transplantation, percutaneous ablation should be considered as the first-line treatment for such patients [124,125,126,127,128]. Ablation can also be proposed as a valid treatment option for BCLC A lesions, given the minimal invasiveness and lower cost of the method in comparison to surgery unless the lesion is in a non-accessible location [2].

One novelty of the new guidelines is the endorsement of treatment stage migration that permits a more flexible approach according to local expertise. Therefore, the indication for ablation might be expanded to all BCLC A cases, even for lesions larger than 3 cm [2]. For the same reason, even patients with up to three lesions that are smaller than 3 cm and who have no transplant option, may undergo ablation in conjunction with TACE.

### 3.2. Rationale for Ablation and Modalities

Ablation offers local tumor destruction either due to chemical or due to energy deposition [126]. It is expected to induce necrosis of the lesion in the specific area where energy is applied and minimizing the damage to the surrounding parenchyma. A safety margin of at least 0.5 cm around the treated lesion is required in order to prevent local recurrence [11].

Energy-based ablation includes radiofrequency ablation (RFA), microwave ablation (MWA), cryoablation (CRA), high-intensity ultrasound ablation (HIFU), laser ablation and irreversible electroporation (IRE). Most of the energy modalities have been used extensively over the years. Among them, RFA has been the most used and studied, even with a “no-touch technique” [127,128,129]. This technique consists of inserting more than one RFA electrode into the periphery of the tumor and performing the ablation after sequential activation of the electrodes. The number of electrodes depends on the size and the geometry of the tumor but usually varies between 2 and 4 [129]. Energy is delivered from the periphery to the center; therefore, the technique ensures that a safety margin is present. However, RFA outcomes are subject to limitations that are mainly related to the location and size of the lesion. Similar modalities such as MWA appear as a very attractive alternative offering larger ablation zones in shorter ablation time and without any heat loss due to surrounding structures.

Cryoablation, on the other hand, is extensively used in the treatment of other lesions such as renal tumors, but has also been introduced in the treatment of HCC in the last few years [130,131]. There are some limitations in the use of cryoablation in the liver. However, this method is also gaining ground in clinical practice. It offers the advantage of concurrent placement of multiple probes and precise intra-procedural monitoring of the created ice-ball. Laser ablation, although rather effective, has not been that extensively used mainly because of the multiple needles required and the relatively smaller ablation zones [132]. On that note, HIFU has also been mainly used empirically and as a bridging treatment for transplant. Another modality that is becoming popular is IRE, a non-thermal energy ablation method that induces cellular apoptosis, based on the irreversible electroporation of cellular membranes. The main advantage of IRE is that it does not cause any damage to the epithelial lining and can be therefore used in more challenging anatomical locations, i.e., close to large vessels or to bile ducts or the gallbladder. However, the cost is still high, general anesthesia and muscular relaxation are required and multiple needles in a precise geometric conformation need to be placed [133].

Nevertheless, in the BCLC criteria, when the term “ablation” is mentioned, this is mainly referring to RFA or MWA. Other ablation techniques like cryoablation and IRE have not been incorporated yet in the published guidelines due to low evidence. Furthermore, in real-life clinical practice, ablation, with or without combined TACE, might be used also for BCLC C patients; however, this is also not included in the guidelines and can be recommended only after multi-disciplinary discussion and if no other treatment option is available.

### 3.3. Outcomes—Complications

The outcomes that measure the performance of ablation are mainly local tumor control, recurrence rate and overall survival. Recurrence rate is expected to be around 70–80% at five years; however, this was shown to be conditioned by systemic treatment, both with sorafenib and immunotherapy [134,135,136]. Potential complications include bleeding, tumor seeding, adjacent organ accidental damage or thermal injury of biliary ducts [11].

### 3.4. RFA vs. MWA

Technically, MWA is expected to offer several advantages over RFA, including faster heating over a larger volume and less susceptibility to heat sink and local perfusion [137]. However, clinical outcomes appear still to be very similar between the two modalities [138]. A comparison between the outcomes of the two percutaneous modalities was published in a meta-analysis on 774 patients [139]. Surprisingly, while complete response rate was marginally higher for MWA, the 3-year survival rate was slightly higher for RFA. MWA obtained better results in terms of local recurrence rate for larger lesions but also led to a higher rate of major complications. Overall, the authors concluded that the two percutaneous modalities offer similar efficiency. However, in the meta-analysis, randomized controlled trials and case-control studies were mixed, when they should have been interpreted separately. On this “eternal debate”, another meta-analysis, published in 2019 on a more extensive population of 1816 patients that were analyzed in 4 randomized control trials and 10 cohort studies, concluded that for the percutaneous treatment the two modalities appear to offer similar therapeutic effect [138]. A third meta-analysis published in 2020 [140] offered the same conclusion for the 3-year follow-up. Perhaps the only paper favoring MWA is that of Bouda et al. [141], where in relation to RFA, a lower local progression rate was found (23% versus 36% for RFA).

### 3.5. Puncture Technique and Navigation Assistance

Ablation can be performed under US- or CT-guidance. Combination of both modalities offer some advantages, as well. CT is not a real-time imaging modality; thus, puncturing a relatively small HCC can be more challenging than with the use of US-guidance, where precise lesion puncture can be made with the needle placed in the middle of the nodule. This is the reason why Hermida et al. propose that US should be the first-line guidance modality for 2–3 cm HCCs [142]. Therefore, the exact needle position can lead to lower local recurrence rate, which is an interesting outcome factor. With US guidance the ablative process can be better followed, while with CT the ablation result can be monitored immediately after the procedure.

Novel navigation systems offer valuable assistance for placing one or more needles in the correct position, especially if HCCs are in difficult positions, such as sub-diaphragmatic locations in the liver dome [143].

### 3.6. Ablation vs. Surgery

The comparison between the two treatment approaches has been previously performed given the very similar indications for the BCLC 0 and BCLC A cases. Indications in everyday clinical practice would favor ablation in presence of portal hypertension with a gradient higher than 10 mmHg or other comorbidities that would contribute towards a more complex surgical approach [144]. However, in the absence of clinically significant portal hypertension, resection should be considered as stated by the recent BCLC criteria [2]. Transplant should also be addressed if microvascular invasion and/or satellite nodules are confirmed after resection [145,146]. Nevertheless, given that the survival rate for lesions smaller than 3 cm appears to be similar if not better for ablation, with lower overall cost and hospital stay, ablation is gaining more ground as first-line treatment for such lesions [124,126,127,128]. For larger lesions, RFA has not performed very well; however, MWA appears to offer better results and is the preferable option if the lesion reaches 4 cm [137].

The location of the lesion also plays a significant role in the clinical decision-making; for intra-parenchymal tumors, ablation may be preferable, whereas if the lesion is close to a thermo-sensitive structure, such as the gallbladder or bowel, then resection might be considered [147]. However, it needs to be taken into account that, with hydro-dissection and use of non-thermal ablation, these boundaries are now reduced. Moreover, in cases of larger HCCs or in location next to major vascular or biliary structures, careful intraoperative ablation is an alternative.

There have been a few randomized trials comparing RFA with resection showing mainly more adverse effects from surgery and a higher recurrence rate from ablation [148,149]. However, this higher recurrence rate of ablation is not conditioning the overall survival [150,151,152,153]. In any case, the complication rate was lower after RFA than laparoscopic resection for small single HCC nodules (5.1 vs.10%) [152].

Cost-effectiveness is also another aspect where ablation prevails [126]. In essence, ablation appears to be superior to surgical resection for HCC up to 3 cm in patients with Child-Pugh class A or B cirrhosis.

### 3.7. RFA vs. Cryoablation

Even though cryoablation is a very promising modality, no clear superiority is shown in the treatment of HCC vs.the other thermal modalities [154]. It needs to be considered that cryoablation is not offering the hemostatic option that both RFA and MWA offer when the electrode is retracted; therefore, provision of the coaxial approach needs to be made, to be in position to embolize the access tract with a hemostatic sponge. Cryoablation also is expected to cause significantly less pain than both RFA and MWA and does not produce the “oven effect,” where heat is trapped within the tumor, as seen in RFA [155].

A multicenter RCT compared the two modalities in 360 patients with Child-Pugh class A or B and up to two lesions of ≥4 cm [156]. Local tumor progression was lower for cryoablation at 3 years (7% vs.11% for RFA); the deference was more pronounced for lesions >3 cm (7.7% vs.18.2%). However, 5-year overall survival, tumor free survival and complication rates were similar between the two modalities. The evidence that cryoablation could offer satisfactory outcomes for lesions larger than 3 cm triggered significant interest on the modality and could potentially change the position of ablation at the BCLC Group HCC management guidelines. However, given the small number of patients, statistical power was subject to condition from the outcome of individual patients.

### 3.8. IRE

Irreversible electroporation offers the advantage of ablating lesions close to vital structures [157]. In a recently published single-center study of patients that could not be treated differently due to the anatomical location of the lesion, local recurrence-free survival at 12 months was 83.6% for a median tumor size of 2 cm [158]. In a comparative study of IRE [159] with RFA and MWA, in terms of complications no significant difference was noticed among thermal and non-thermal modalities even though more needles are required with IRE and no tract cauterization may be performed. Another interesting aspect as suggested by few recent publications is that IRE could be more suitable for patients with cirrhosis [160,161,162,163].

### 3.9. Ablation and Immunotherapy

Immunotherapy appears to be very promising in the treatment of HCC. Ablation is the process of releasing tumor-associated antigens that enhance the immune response against the tumor itself [164]. In a study of 32 patients that underwent treatment with tremelimumab followed by ablation, intratumoral accumulation of CD8+ T cells was detected [165]. There are multiple open questions about the timing of ablation and immunotherapy, e.g., if tumor ablation must be complete or partial and many more [165,166]. Therefore further research is required. Clinical trials investigating the role of ablation on HCC and that are currently recruiting are shown on Table 3.

### 3.10. Prediction Models and Artificial Intelligence

Attempts for predictive models after RFA have been developed in the effort to standardize the approach and improve outcomes. In a recently published study on 238 patients that underwent ablation for early-stage HCC, several factors, i.e., tumor size and a-fetoprotein levels, were related to ablation outcomes [143]. Outcome-predictive models with the use of artificial intelligence have also been developed. In a study of 83 HCC patients, who received ablation as first treatment, features that would predict the outcome were analyzed via five different feature-selection methods [167]. In another study of 252 patients who received RFA, artificial neural network models among 15 clinical variables were created to predict 1- and 2-year disease-free survival, achieving an acceptable prediction performance [168].

## 4. Stereotactic Body Radiation Therapy (SBRT)

### 4.1. Background and Biological Rationale

Through technical advances in the field of radiation therapy, SBRT has become an alternative regional treatment modality for patients with HCC in various stages. The rationale behind SBRT is that it induces DNA damage leading to an inhibition of the cancer cell replication [169]. However, radiation therapy in the treatment of liver cancer was limited only to patients with a very high tumor burden and individualized treatment concepts as the radiation led to great damage in the tumor-surrounding liver parenchyma. With improvements in image guidance and conformal radiation techniques, nowadays, radiation can be applied more precisely to the tumor tissue in high doses, while sparing the surrounding liver tissue [170]. Compared to other local and regional treatment options, SBRT could be beneficial in complex anatomical situations and a high local tumor burden.

A rare complication of SBRT is the radiation-induced liver disease (RILD). RILD is associated with cholestasis, hepatomegaly, increase liver enzymes, impairment of the remaining liver function and development of ascites [171,172]. RILD typically appears in the first 2 months after radiation therapy, but reports vary between 2 weeks and 7 months for the appearance of typical symptoms [173]. To lower the risk for a post-interventional RILD, liver function has to be evaluated carefully prior to SBRT.

### 4.2. Patient Selection: SBRT in Early Stages

In this stage, SBRT is a potential treatment option for patients not suitable for liver resection or transplantation due comorbidities and not suitable for ablation due to lesions near to liver vessels, biliary structures and adjacent organs [174]. Several phase I and II trials have shown promising results for patients with few HCC lesions not suitable for ablation [174]. In these studies, SBRT led to high local tumor control rates. Additionally, larger phase I and II trials showed that SBRT could be applied for bridging to transplant [168]. However, the number of available results from phase III RCTs in patients with early-stage HCC is scarce. In one available non-inferiority study, Kim et al. observed no differences in the local PFS for patients with recurrent HCC compared to RFA [175]. In a second available phase III study, patients with HCC and PVTT undergoing surgery, who had neoadjuvant radiation therapy, had a significantly better postoperative overall survival than patients that did not underwent adjuvant therapy [176]. Although these results for selected patients were promising, more evidence is needed prior clear treatment recommendations can be made.

### 4.3. Patient Selection: SBRT in Intermediate Stage

As the intermediate stage is defined as a group of patients with a high local tumor burden, SBRT has a large potential for improving local tumor control rates compared to other treatment modalities. Several retrospective studies as well as a few phase I and II trials demonstrated high local tumor control rates [174]. Up to date, however, no phase III trial results comparing SBRT to TACE are available. A recent meta-analysis consisting of ten retrospective studies showed a higher complete response rate as well as longer overall survival for patients receiving combined TACE and SBRT compared to patients receiving only SBRT [177].

### 4.4. Patient Selection: SBRT in Advanced Stages

Several retrospective studies and a few phase I and II trials have shown promising results for SBRT in patients with advanced HCC and macroscopic vascular invasion or impaired liver function [174]. However, phase III trials comparing SBRT with standard systemic treatment are currently not available. Only for the combined treatment of TACE and radiation therapy, a recent RCT reported superior outcome results including a significantly prolonged overall survival for patients with HCC and macrovascular invasion compared to a treatment with sorafenib [82]. Nevertheless, the role of SBRT compared to systemic treatment and particularly to the novel standard treatment combining atezolizumab and bevacizumab remains unclear.

### 4.5. SBRT Combined with Other Treatment Modalities

As mentioned above, the combination of TACE and SBRT has a high potential for patients with unresectable HCC, despite the fact that evidence for the combination of SBRT and TARE is low. Several retrospective analyses and phase I trials have yielded promising results for the combined use of sorafenib and SBRT [81], while a phase III trial of the Radiation Therapy Oncology Group is currently recruiting (NCT01730937). In this context, preclinical investigations have demonstrated the potential of sorafenib to function as a radiosensitizer [81]. The same seems to be apparent for immunotherapeutic agents. Vice versa, local radiotherapy might interfere with the immune reaction of the tumor microenvironment leading to immunogenic cell death [81]. Thus, radiation and immunotherapy could function as a strong synergistic treatment. However, up to date evidence is low and only a few small retrospective studies have been published so far [178]. Several prospective phase I and II studies investigating the combination of SBRT and various immunotherapeutic agents in various patient subgroups are currently recruiting [81].

### 4.6. Current Recommendations

Current guidelines show a significant disparity in terms of their recommendations for SBRT. While the current EASL guideline does not recommend SBRT for patients with HCC due to the low amount of evidence, the currently updated BCLC classification does not even mention SBRT as treatment option. Contrary, the European Society for Medical Oncology (ESMO) as well as the American Association for the Study of Liver Diseases (AASLD) guidelines do recommend radiation therapy as a treatment option for selected patients with HCC [1,2,101,179]. However, these guidelines also argue for a need of more evidence before stronger recommendations can be made. In contrast to these rather soft recommendations, the American Society for Radiation Oncology (ASTRO) has recommended the use of SBRT strongly for patients within the early stages for whom surgery and ablation is no treatment option [180]. Furthermore, the ASTRO guideline recommends a sequenced use of SBRT for patients with multifocal, unresectable HCC, in patients with macrovascular tumor invasion and for relief of symptoms in the palliative setting of best supportive care. Thus, the range of recommendations is significantly different between the various guidelines. It remains to be seen how the recommendations of the different societies will adapt to the results of the increasingly available RCTs results with the upcoming updates of the guidelines.

## 5. The Potential of Combined Treatment—Current State and Future Directions

In this article, we aimed to summarize the current state of locoregional HCC treatment with a special focus on combined treatment options. From our point of view, an important aspect for the future of HCC treatment is a better understanding of how we can combine locoregional and systemic treatment. While expert knowledge and techniques for local and regional treatment options have continuously increased over the last decade, immunotherapy has had a rapid rise in the recent years and opened new doors for treatment of more advanced stages. In the context of combined treatment, immunotherapy has the potential to compensate various drawbacks of local and regional treatment modalities, which have been mentioned above. Furthermore, compared to previous studies combining locoregional treatment with tyrosine kinase inhibitors, the combination with immunotherapeutic agents offers a significantly improved side-effect profile. Thus, these novel options have the potential to facilitate HCC treatment towards highly individualized treatment approaches in the context of personalized medicine. To enable individualized treatment approaches, the identification of patients likely to benefit from such combined approaches remains of the utmost important. Although the knowledge on predictive biomarkers in patients with HCC and immunotherapy is continuously investigated, the current knowledge is still limited and, for combined locoregional and immunotherapeutic treatment, evidence on predictive biomarkers is missing completely. One fact attributing to the lack of biomarkers is the limited number of patients currently treated with combined locoregional and immunotherapeutic treatment. However, with the ongoing of the above-mentioned trials investigating various combinations, potential collectives for biomarker evaluation will be available.

## 6. Limitations

With this review, we aimed to give the reader a comprehensive overview on the aspects of utmost importance when evaluating local and regional treatment options in patients with HCC undergoing TACE. This review article particularly focused on the potential of combined treatment options. Of course, our review has the typical drawbacks of an expert review that need to be addressed. First, the structure of the article as well as the recommendations and literature selection were biased by the subjective estimations of the authors. Secondly, this review is not a systematic review. Thus, some relevant articles could have been missed. Thirdly, expert reviews always have a potential bias of authors interpreting the original data leading to different conclusions and recommendations. One important aspect that has to be mentioned is that patient selection in the cited retrospective or single-arm studies were based on very heterogeneous inclusion criteria, and the patient cohort varied strongly. Thus, a strong influence of the inclusion bias has to be estimated and, particularly, reported survival results should not be generalized.

## 7. Conclusions

In summary, local and regional therapies remain a mainstay of curative and palliative treatment of patients with HCC. Currently, evidence on potential combination of the local and regional treatment options with each other as well as with other treatment modalities is growing and has the potential to further individualize HCC therapy. To identify the most suitable treatment out of these new various options, a repeated interdisciplinary discussion of each case by the tumor board is of the utmost importance.

## Figures and Tables

**Table 1:** Overview on currently applied immune- and inflammation based prognostic indices in patients with HCC undergoing TACE. Modified according to [57].

Index	Concept and Characteristics	Included Parameters	Pros	Cons	Current Research Status
NLR	-captures shifts in the relationships between blood cells, due to immune response effects	-neutrophil count -lymphocyte count	-simple calculation -well investigated	-nutritional status not included -divergent results in studies that compared NLR to other immune-based indices	-designed for the stratification of critically ill patients, and validated in patients with colorectal cancer, in an oncologic context [63] -extensively validated for various cancer entities, including patients with HCC undergoing TACE
PLR	-captures shifts in the relationships between blood cells, due to immune response effects	-platelet count -lymphocyte count	-simple calculation -well investigated	-nutritional status not included -divergent results in studies that compared PLR to other immune-based indices	-designed for the stratification of patients with pancreatic cancer [64] -extensively validated for patients with HCC undergoing TACE
CALLY	-combines inflammation, immune response, and nutritional status markers (aspects of the PNI) -for liver disease, albumin functions as an indicator of liver function	-CRP -albumin -lymphocyte count	-novel combination of inflammation, immune response, nutritional status, and liver function markers provides a more holistic assessment	-CALLY was not superior to previously established scoring systems	-designed for a cohort of patients with HCC undergoing resections [65] -only validated in one study for patients with HCC undergoing TACE
PNI	-combines immune response and nutritional status markers	-albumin -lymphocyte count	-combination of immune response and nutritional status markers	-few studies available on patients with HCC undergoing TACE -divergent results regarding the predictive ability of PNI -the mathematical calculation may require improvement	-designed for patients with gastric cancer [66] -extensively validated for various cancer entities -few studies available for patients with HCC undergoing TACE -divergent results on its predictive ability -PNI combined with ALBI was identified as a novel, feasible stratification system for patients with HCC undergoing TACE [55]
CONUT	-combines immune response and nutritional status markers	-albumin -lymphocyte count -cholesterin	-combination of immune response and nutritional status markers	-few studies available on patients with HCC undergoing TACE -not superior to PNI [56]	-Only few validation results in patients with HCC undergoing TACE
SII	-combines inflammation and immune response markers	-lymphocyte count -neutrophil count -platelet count	-extensively validated for patients with HCC	-nutritional status not included - literature is scarce for patients undergoing TACE	-designed for the stratification of patients with HCC undergoing resections [67] -extensively validated for various cancer entities -few studies on the role of the SII in patients undergoing TACE
ILIS	-combines inflammation, liver function, and tumor markers -specifically developed for patients with HCC	-albumin -bilirubin -alkaline phosphatase -neutrophil count	-index is specific for HCC -includes tumor and liver function markers	-complex calculation -scarce literature for patients with HCC, particularly for patients undergoing TACE	-specifically designed for patients with HCC [68] -only one external validation study available

**Table 2:** Overview on ongoing or planned randomized clinical phase 2/3 trials currently investing the combination of TACE and immunotherapeutic agents.

Trial Name	Identifier	Phase	BCLC Stage	Treatment Arms	Primary Endpoint(s)
LEAP-012	NCT04246177	Phase 3	B	Lenvatinib + pembrolizumab + TACE TACE alone	PFS per RECIST1.1 OS
EMERALD-1	NCT03778957	Phase 3	B	Durvalumab + TACE Durvalumab + bevacizumab + TACE TACE alone	PFS per RECIST1.1
CheckMate 74W	NCT04340193	Phase 3	B	Nivolumab + ipilimumab + TACE Nivolumab + TACE TACE alone	Time to unTACEble progression OS
TACE-3	NCT04268888	Phase 2/3	B	Nivolumab + TACE/TAE TACE/TAE alone	Time to unTACEble progression OS
TALENTACE	N/A	Phase 3	B	Atezolizumab + bevacizumab + TACE TACE alone	TACE-PFS OS

**Table 3:** Overview on currently recruiting clinical trials investing the use of ablation for the treatment of HCC.

Trial Name	Identifier	Phase	BCLC Stage	Treatment Arms	Primary Endpoint(s)
IMMULAB	NCT03753659	Phase 2	A	Pembrolizumab + RFA/MWA/brachytherapy or TACE	ORR per RECIST1.1
	NCT04663035	Phase 2	A	Ablation + Tislelizumab vs. Ablation Alone	RFS
AB-LATE02	NCT04727307	Phase 2	A	RFA + Atezolizumab + Bevacizumab vs. RFA Alone	RFS
	NCT04652440	Phase 2	A/B	RFA + Tislelizumab	TRAEs SAEs Tolerability
	NCT02964260	Phase 2	B	TAE + Ablation (simultaneously) TACE + Ablation (1 month interval)	OS
	NCT04365751	N/A	B	MWA vs. Laparoscopic hepatectomy	OS
	NCT03898921	3	A/B	SBRT vs. RFA	OS
	NCT04220944	1	B/C	MWA + TACE + Sintilimab	PFS

ORR—objective response rate; TTR—time to recurrence; RFS—recurrence-free survival; TRAEs—treatment-related adverse events; SAEs—serious adverse events; OS—overall survival; SBRT—Stereotactic Body Radiotherapy.
